# Preparation and Characterization of Tadpole- and Sphere-Shaped Hemin Nanoparticles for Enhanced Solubility

**DOI:** 10.1186/s11671-019-2880-7

**Published:** 2019-02-06

**Authors:** Jie Yang, Liu Xiong, Man Li, Junxia Xiao, Xin Geng, Baowei Wang, Qingjie Sun

**Affiliations:** College of Food Science and Engineering, Qingdao Agricultural University, 700 Changcheng Road, Chengyang District, Qingdao, 266109 China

**Keywords:** Fabrication, Soluble, Nanoparticles, Stability, Hemin

## Abstract

Hemin is a potent iron supplement. A major limitation of the applicability of hemin is its extremely low aqueous solubility and bioavailability. The aim of this work is to prepare hemin nanoparticles with improved solubility. Tra*nsmission electron microscopic images showed that hemin nanoparticles with different initial concentrations* of hemin (0.1 and 0.5 mg/mL) were tadpole-shaped (head of approximately 200 nm and tail of 100 nm) and sphere-shaped (50–100 nm), respectively. Moreover, hemin nanoparticles exhibited higher solubility than free hemin. The solubility of sphere-shaped nanoparticles was 308.2-fold higher than that of pure hemin at 25 °C. The hemin nanoparticles were stable in acidic conditions and displayed excellent thermal stability. These results suggested that hemin nanoparticles could serve as a potential iron supplement with potential applications in the food, biomedical, and photodynamic-photothermal therapy fields.

**Electronic supplementary material:**

The online version of this article (10.1186/s11671-019-2880-7) contains supplementary material, which is available to authorized users.

## Introduction

Iron is an essential element in the body’s metabolic processes, such as electron transfer, storage, and oxygen transport [1]. Iron deficiency is one of the most common nutritional deficiencies, influencing roughly 20% of the world’s population [2]. The most negative consequence of iron deficiency is sideropenic anemia. It is mostly caused by insufficient dietary intake of iron, often when the demand is high. In humans, dietary iron can enter the body in two main forms: one is inorganic iron (nonheme iron), mainly released from vegetables and plant-based foods, and the other is heme iron, acquired from the breakdown of hemoglobin or myoglobin present in animals. Hemin has been found in blood and meat products, is a component of many hemoproteins (including myoglobin, hemoglobin, and cytochromes b and c), and is two- or threefold more easily absorbed (50–87%) than nonheme iron [3]. Recently, researchers have witnessed major advances in our understanding of the physiological role of hemin. Unfortunately, hemin is hydrophobic due to the presence of a large tetrapyrrolic macrocycle [4]. Due to the high hydrophobicity and poor solubility of hemin in neutral aqueous solution, its application in various fields has been limited. Thus, there is an urgent need to increase the solubility of hemin.

To address this challenge, many efforts have been devoted to improving the solubility of hemin. Berner [5] discovered that partial enzyme solution protein (soy isolate, soy flour, or soy concentrate) could unite with heme iron to enhance iron absorption, which improved iron bioavailability. Wang et al. [6] indicated that crystalline hemin and L-arginate could prepare water-soluble hemin-arginate coacervation, which could be used as a new heme iron supplement in food additives, functional foods, and pharmaceuticals. Zhang et al. [7] reported that hemin could combine with β-cyclodextrin by a cyclic oligosaccharide of seven α- linked glucose units [1, 4], leading to a significant improvement in hemin solubility. Although there was some progress on the solubility improvement of heme iron, industrialization was not easy due to the complicated preparation process. Hence, developing a simple method to improve the solubility of hemin is still a major challenge.

Nanoscience and nanotechnology have the potential to provide new solutions in the development of functional substances, particularly the inclusion of bioactive compounds without affecting the sensory perception of consumers and improving the uptake of certain components [8]. Nanoparticles have several advantages [9], including promoting the solubility of hydrophobic substances [10]. Duhem et al. [11] developed novel vitamin E-based nanomedicines through nanotechnologies, which offered multiple advantages in drug delivery like biocompatibility, improved drug solubility, and anticancer activity. Chang et al. [12] reported that the nanoparticles prepared by succinic anhydride-modified short glucan chains could load hydrophobic lutein, which could enhance the water solubility of lutein. Despite the huge potential of nanoparticles, nanoscale hemin has yet to be reported. We postulated that the solubility of hemin nanoparticles could be increased compared to free hemin, which could have valuable applications.

The primary objective of the present work is to develop different-shaped hemin nanoparticles using a facile dialysis technique and to enhance their solubility. The preparation parameters of the initial hemin concentration and dialysis conditions were evaluated. Additionally, the solubility and stability against the pH, thermal treatment, and salt of the formed hemin nanoparticles were assessed. Overall, improvements in the solubility of hemin have a variety of potential application fields.

## Materials and Methods

### Materials

Hemin and dialysis membranes with a molecular weight cutoff of 8–12 kDa were purchased from Beijing Solarbio Science & Technology Co., Ltd. (Beijing, China). Acetone (CH_3_COCH_3_, ≥ 99.5%) was procured from Kant Chemical Co., Ltd. (Laiyang, China). All other reagents used were of analytical grade.

### Preparation of Hemin Nanoparticles

Hemin nanoparticles were prepared using a dialysis method: 0.1 mg/mL (or 0.5 mg/mL) of hemin dissolved in acetone acidified with 0.1 ml of concentrated hydrochloric acid. The hemin solution was dialyzed for different days, the water was changed every day, and it was lyophilized to obtain hemin nanoparticles. To ascertain the effect of variable parameters on the preparation of hemin nanoparticles, the hemin/water volume ratios were set at 1:3, 1:5, 1:10, and 1:50; the cultivate temperatures were set at 4 and 25 °C; and the incubation times were set at 1, 3, and 5 days.

### Transmission Electron Microscopy (TEM)

TEM images of nanoparticles were taken with a 7700 transmission electron microscope (Hitachi, Tokyo, Japan) with an acceleration voltage of 80 kV. A tiny sample drop was deposited on a carbon-coated copper grid, then freeze-dried for observation.

### Average Size and Zeta Potential Measurements

The average size, zeta potential (ζ-potential), and polydispersity index (PDI) of particles were measured via dynamic light scattering (DLS), using a Malvern Zetasizer Nano (Malvern Instruments Ltd., UK). The samples were diluted in MilliQ water and analyzed at 25 °C. The concentration of diluted samples was 0.05%.

### UV–Vis Absorption Spectrum

UV–Vis spectroscopy measurements of the free hemin and hemin nanoparticles dissolved in acidified aqueous acetone were carried out on a UV–Vis spectrophotometer (TU-1810, Beijing, China). The molecular absorption was scanned at a wavelength of 200–800 nm at 1-nm intervals to obtain a spectrum.

### Solubility Assay

The quantitative aqueous solubility of pure hemin and nanoparticles was investigated according to the method reported by Gidwani et al. [13]. Briefly, supersaturated solutions of pure hemin and nanoparticles were added separately to 5 ml of deionized water in test tubes, respectively. The test tubes were stirred constantly (500 rpm) at different temperature (25, 37, 60, and 80 °C) for 30 min. Then, the solution was centrifuged at 3500*g* and supernatant was diluted suitably with acidified aqueous acetone. The concentration of samples was determined at 640 nm by a UV–Vis spectrophotometer. For each measurement, the baseline was established using blank acidified aqueous acetone as a reference.

### pH, Temperature, and Salt Stability

The size, ζ-potential, PDI, and turbidity of the nanoparticles (0.5 mg/mL) were measured and compared with the initial values to evaluate the stability of the nanoparticles. The particle suspensions were divided into ten groups: six groups were adjusted to the desired pH values [2, 3, 5, 7, 9, 11], and using hydrochloric acid (0.1 M) or sodium hydroxide solution (0.1 M); three groups were heated to 25, 60, and 80 °C and then cooled to room temperature; another was conducted with different concentrations of sodium chloride (NaCl, 0, 10, 50, 100, 250, and 500 mM), respectively. The mixed solutions stood overnight at 25 °C.

### Fourier Transform Infrared (FTIR) Spectroscopy

The chemical structures of hemin nanoparticles were confirmed using FTIR spectra (Tensor 27, Jasco Inc., Easton, MD, USA). A total of 32 scans at a resolution of 4 cm^− 1^ were accumulated using rapid-scan software in OMNIC 8.0 to obtain a single spectrum. The spectral range was 400–4000 cm^− 1^.

### Fluorescence Spectroscopy

Fluorescence measurements of free hemin and nanoparticles were performed using a fluorescence spectrophotometer (F-7000, Hitachi, Japan). The fluorescence spectra of the samples were obtained at wavelengths between 300 and 600 nm with excitation at 402 nm.

### X-Ray Diffractogram (XRD)

The XRD of free hemin and nanoparticles was obtained using an X-ray diffractometer (AXS D8 ADVANCE; Bruker, Karlsruhe, Germany), and the samples were investigated in the 2*θ* range of 4–40°. The relative crystallinity of free hemin and nanoparticles was determined by plotting baseline of the peaks on the diffractogram and calculating the area using the software spectrum viewer based on the method reported by Jivan et al. [14]. The area above and under the curve corresponded to crystalline domains and amorphous regions, respectively. The ratio of the upper area to the total area was taken as the relative crystallinity:

Relative crystallinity (%) = Area under the peaks/Total curve area × 100.

### Statistical Analysis

Triplicate samples of all quantitative results were obtained. The results were reported as the average values and standard deviations. Statistical analysis was performed by Duncan’s multiple range tests using the SPSS V.17 statistical software package (SPSS Inc., Chicago, IL, USA).

## Results and Discussion

### Formation and Characterization of Hemin Nanoparticles

**Fig. 1:**
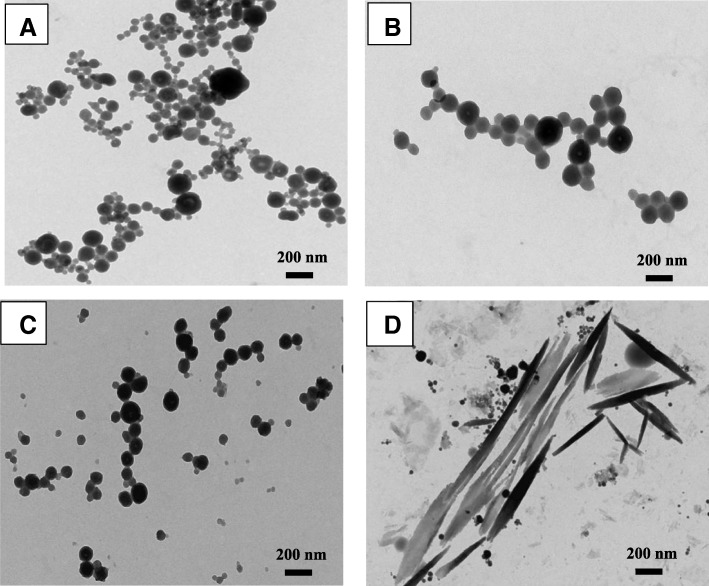
TEM images of hemin nanoparticles prepared by dialyzing for 3 days with various hemin/water volume ratios, including 1:3 (**a**), 1:5 (**b**), 1:10 (**c**), and 1:50 (**d**) at 25 °C. The concentration of hemin was 0.5 mg/mL

The morphology and size of hemin nanoparticles fabricated using the dialysis method were examined by TEM. When the hemin concentration was 0.5 mg/mL, hemin nanostructures with different sizes were formed at various hemin/water volume ratios and for different dialysis days (Fig. 1, Additional file 1: Figure S1–S3). The nanoparticles had well-defined spherical shapes and had diameters of 50–100 nm when the hemin/water volume ratio was 1:10 after 3 days of dialysis. With an increase in the hemin/water volume ratio (1:50), nanoparticles were gathered into rod-shaped particles (Fig. 1). Particularly, we found that as the dialysis time increased from 1 to 3 days, the hemin nanoparticles became uniformly dispersed (Additional file 1: Figure S1-S3). The temperature (4 and 25 °C) of the dialysis had little influence on the particle size and the dispersion of hemin nanoparticles (Additional file 1: Figure S4).

**Fig. 2:**
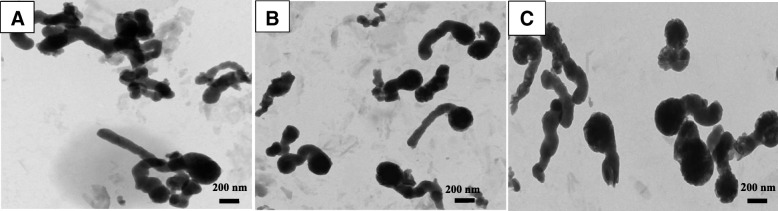
TEM images of hemin nanoparticles prepared with the hemin/water volume ratio of 1:10 at various dialysis days including 1 day (**a**), 3 days (**b**), and 5 days (**c**) at 25 °C. The concentration of hemin was 0.1 mg/mL

Figure 2 shows the typical TEM images of hemin nanoparticles prepared for different dialysis days with the hemin concentration of 0.1 mg/mL. The products were mainly well-defined, singly dispersed, structurally unusual, and tadpole-shaped nanoparticles. The tadpole-like nanoparticles were preferably distributed with 3 days of dialysis. The tadpole displayed a significant disparity in size from the maximum width of the head (200 nm) to the tail (100 nm). Our results showed that uniformly dispersed nanoparticles could be formed at the hemin/water volume ratio of 1:10 and after dialyzing for 3 days at 25 °C for both sphere- and tadpole-shaped nanoparticles. Thus, we chose these two kinds of hemin nanoparticles for the following research.

**Fig. 3:**
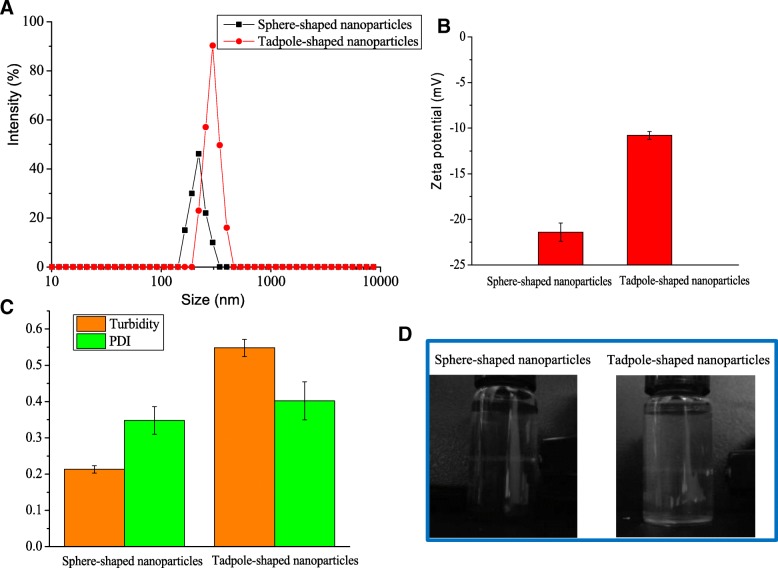
The average size (**a**), ζ-potential (**b**), PDI and turbidity (**c**), and Tyndall effect (**d**) of different-shaped hemin nanoparticles

To further examine the size of hemin nanoparticles, DLS determination was used to confirm the formation of nanostructures. The diameters of sphere- and tadpole-shaped nanoparticles were approximately 218.2 ± 6.2 and 299.8 ± 7.6 nm, respectively (Fig. 3a). The size of the nanoparticles measured by DLS was somewhat larger than the results measured by TEM; this difference was attributed to the nanoparticles swelling in aqueous solution. The DLS measurement was known to indicate the hydrodynamic diameters of nanoparticles in a solution [15]. The ζ-potential of sphere-shaped nanoparticles (− 21.4 mV) was approximately twice as high that of tadpole-shaped nanoparticles (− 10.8 mV) (Fig. 3b). The PDI of the hemin nanoparticles were also determined to analyze the particle size distribution. The results showed that the PDI of the sphere-shaped and tadpole-shaped nanoparticles was 0.348 and 0.402, respectively (Fig. 3c). This finding indicated that the obtained hemin nanoparticles had good polydispersity.

As a light beam passes through a colloidal dispersion, a portion of light is scattered by the colloidal particles present in the solution, leading to a divergence of the light beam. This behavior is called the Faraday-Tyndall effect [16]. In this case, the free hemin solution did not present a Tyndall effect (Additional file 1: Figure S5). Nevertheless, the Tyndall effect was observed in the suspension of both sphere- and tadpole-shaped nanoparticles (Fig. 3d), verifying the formation of colloids or nanoparticles in the fine suspension. The formation mechanism of hemin nanoparticles by the dialysis method may be due to diffusion of solvent through the interface between water phase outside and organic solvent phase inside, which resulted in a decrease in solubility of hemin and the formation of crystal nucleus. Subsequently, different-shaped hemin nanoparticles were formed due to the growth and self-assembly of single crystal nucleus in different manners.

### UV–Vis Absorption Analysis

**Fig. 4:**
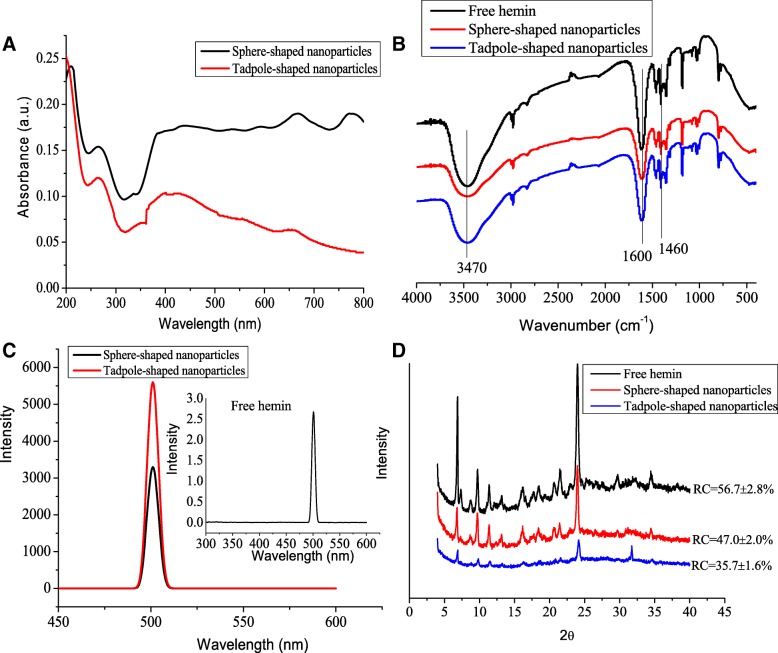
**a** UV–Vis spectrum, **b** FTIR spectra, **c** fluorescence emission spectra, and **d** XRD patterns of free hemin, sphere-shaped nanoparticles, and tadpole-shaped nanoparticles. The nanoparticles were dispersed in deionized water. RC, relative crystallinity

According to the above results, we investigated whether there were any differences between the two shapes of hemin nanoparticles in their chromophore generation. The UV–Vis spectrum of both sphere- and tadpole-shaped nanoparticles showed the same absorption bands at 265 nm (Fig. 4a). The sphere-shaped nanoparticles presented a narrow absorption band at 667 nm and 775 nm. In comparison, the tadpole-shaped nanoparticle solution showed a broad peak at 658 nm without a peak of 775 nm. Moreover, the absorption intensity of sphere-shaped nanoparticles was higher than that of tadpole-shaped nanoparticles. Such a large difference could neither be explained by regarding the tadpole as a sum of a sphere and a tapered rod nor by a somewhat imperfect sphere configuration. The electron oscillation corresponding to a plasmon absorption along the long axis is retarded and/or on a reflective path. Thus, the optical properties of hemin nanoparticles were dependent on shape, similar to the report by Hu et al. [17], who found that tadpole- and sphere-shaped gold nanoparticles had different optical properties.

The free hemin solution displayed a maximum absorption at 344 nm, and this was attributed to the Soret band commonly associated with porphyrins (Additional file 1: Figure S5). The absorption band of hemin nanoparticles shifted from 344 to 265 nm, which suggested that the π-π conjugative effect of hemin nanoparticles was enhanced. Surprisingly, in comparison with free hemin, both kinds of nanoparticles exhibited a high, strong near-infrared absorption, which is highly appropriate for absorption-based applications, such as photothermal therapy and photoacoustic imaging [18]. Magno et al. [19] also reported that porphyrin nanoparticles with near-infrared absorption have received considerable interest for applications in phototherapies and photodiagnostics, even as magnetic nanoparticles for magnetic-hyperthermia therapy and drug-delivery systems.

### Solubility

The solubility of hemin is an important factor, which can directly affect the absorption efficacy in the body. The quantitative aqueous solubility of pure hemin and hemin nanoparticles at different temperatures were tested (Table 1). As the temperature increased, the aqueous solubility of all the samples increased. For example, the solubility of free hemin at 25, 37, 60, and 80 °C was 0.009 ± 0.000, 0.060 ± 0.002, 0.144 ± 0.004, and 0.245 ± 0.008 mg/mL, respectively (Table 1).

**Table 1:** Solubility of free hemin and hemin nanoparticles at different temperatures

Sample	Solubility (mg/mL)			
	25 °C	37 °C	60 °C	80 °C
Free hemin	0.009 ± 0.000c	0.060 ± 0.002c	0.144 ± 0.004c	0.235 ± 0.008c
Sphere-shaped nanoparticles	1.333 ± 0.023a	1.499 ± 0.072a	1.889 ± 0.081a	3.853 ± 0.124a
Tadpole-shaped nanoparticles	0.997 ± 0.045b	1.231 ± 0.035b	1.521 ± 0.058b	1.795 ± 0.050b

The values correspond to the mean ± standard deviation. Values within each column followed by different letters indicate significant differences (*P* < 0.05)

The amount of sphere-shaped nanoparticles dissolved at 25, 37, 60, and 80 °C was 1.333 ± 0.023, 1.499 ± 0.072, 1.889 ± 0.081, and 3.853 ± 0.124 mg/mL, respectively, and that of tadpole-shaped nanoparticles were 0.997 ± 0.045, 1.231 ± 0.035, 1.521 ± 0.058, and 1.795 ± 0.050 mg/mL, respectively. The results of the aqueous solubility study of nanoparticles showed a significant increase in comparison to pure hemin. The sphere-shaped nanoparticles exhibited higher solubility at temperatures of 25, 37, 60, and 80 °C than those of the tadpole-shaped nanoparticles. This finding suggests that the solubility of the sphere-shaped nanoparticles could be 308.2-fold higher compared to free hemin at 25 °C. This increase in solubility was mainly due to the unique nanoscale particle size. This result was consistent with other studies reported by Gidwani and Vyas [13].

### FTIR Spectra Analyses

FTIR spectra can be used to identify types of functional groups. The band at 3470 cm^− 1^ is mainly attributed to the stretching vibration of N–H and hydroxyl groups from hemin (Fig. 4b). A band at 1460 cm^− 1^ is ascribed to the N–H in-plane vibration due to the out-of-plane bending vibration of –CH_3_ from the aromatic pyrrole ring of hemin. The peak at 1600 cm^− 1^ is the characteristic peak of the amide bond owing to the stretching vibration of C=O of the surface bound carboxyl group of hemin, which reveals that the secondary amide bond exists in hemin. These results are consistent with those of Xi et al. [20]. However, the peak at 3470 cm^− 1^ of hemin nanoparticles was broader than that of free hemin, clearly indicating the enhanced hydrogen bonding interaction between nanoparticles.

### Fluorescence Spectra

The fluorescence properties of free hemin and hemin nanoparticles were also monitored by fluorescence spectroscopy. The fluorescence signals of both sphere- and tadpole-shaped nanoparticles were increased in the apparent emission maximum at 500 nm compared to free hemin (Fig. 4c). This could be due to the increased solubility of hemin after the formation of nanoparticles [21].

### XRD Analysis

The crystalline nature of free hemin and hemin nanoparticles was confirmed by XRD. As illustrated in Fig. 4d, the XRD patterns of free hemin displayed several relatively strong reflection peaks at 2*θ* = 6.8, 9.6, 11.5, 16.2, 21.5, and 23.9°. The characteristic peaks of the sphere-shaped nanoparticles were the same as those of free hemin, thus indicating that the crystal structure of sphere-shaped nanoparticles did not change in the nanoparticle formulations. However, for the tadpole-shaped nanoparticles, most characteristic peaks disappeared. In addition, the relative crystallinity of sphere- and tadpole-shaped nanoparticles was significantly decreased to 47.0% and 35.7%, respectively, compared to 56.7% for free hemin. These results indicated that the nanoparticle formulations could destroy the partial crystalline regions of hemin.

### Effects of pH, Temperature, and Salt Concentration on Stability

**Fig. 5:**
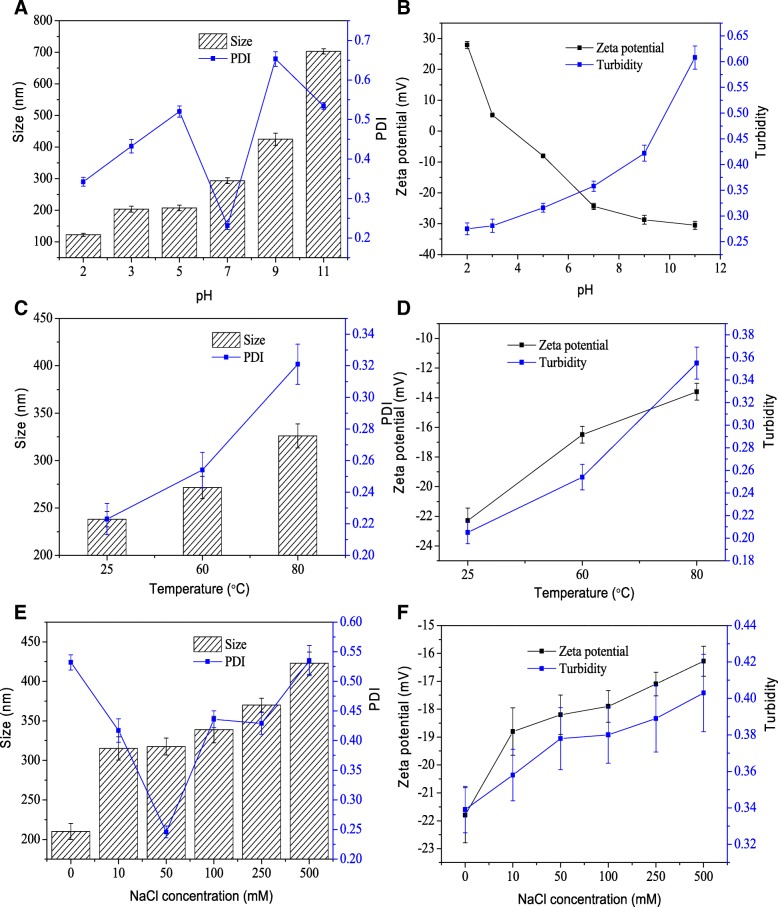
Stability of hemin nanoparticles. The effect of various pH levels (**a**), temperatures (**c**), and salt concentrations (**e**) on the particle size and PDI of nanoparticles. The effect of various pH levels (**b**), temperatures (**d**), and salt concentrations (**f**) on the ζ-potential and turbidity

The variations in the size, PDI, ζ-potential, and turbidity of hemin nanoparticles after incubation at different pH levels [2–11] were measured (Fig. 5a, b). The size of hemin nanoparticles remained almost unchanged with average diameters of approximately 200 nm under acidic conditions (Fig. 5a). At low pH values of 2, the size of the hemin nanoparticles was decreased to approximately 122.4 nm. At a pH of 7, the size of the nanoparticles increased to 293.6 nm, and it increased significantly (*P* < 0.05) under alkaline conditions (a pH of 9 and 11.0) with average diameters over 400 nm. The PDI of the nanoparticles was less than 0.5 in acidic conditions, demonstrating no apparent aggregation of nanoparticles [22]. The ζ-potential of nanoparticles decreased with increasing pH values (Fig. 5b). The turbidity of nanoparticles showed the same trend in size. These results indicated that nanoparticles were stable in acidic conditions and unstable in alkaline conditions.

The effects of thermal treatment (25, 60, and 80 °C) for 30 min on the size, PDI, ζ-potential, and turbidity of hemin nanoparticles were determined (Fig. 5c, d). When the temperature was increased, the particle size, PDI, ζ-potential, and turbidity of nanoparticles slightly increased. The results suggest that the hemin nanoparticles had excellent thermal stability. Similarly, as the ionic strength was increased, the size, ζ-potential, and turbidity of nanoparticles were also increased, which caused the dissociation of the nanoparticles (Fig. 5e, f).

## Conclusions

In this work, we first developed tadpole- and sphere-shaped hemin nanoparticles using a facile dialysis technique, which could significantly enhance the solubility by 308.2-fold at 25 °C. Moreover, the hemin nanoparticles were stable in acidic conditions and displayed excellent thermal stability. In addition, both nanoparticles exhibited strong near-infrared absorption. Future work will focus on the in-depth study of the design of an optothermal response hemin nanocarrier system for active ingredient loading. Hemin nanoparticles with enhanced solubility could have potential applications in the biomedical, food, photodynamic therapy, and photodynamic-photothermal therapy fields.

## Additional file

Additional file 1: **Figure S1.** TEM images of hemin nanoparticles prepared by dialyzing for one day with various hemin/water volume ratios, including 1:3 (A), 1:5 (B), 1:10 (C), and 1:50 (D) at 25 °C. The concentration of hemin was 0.5 mg/mL. **Figure S2.** TEM images of hemin nanoparticles prepared by dialyzing for two days with various hemin/water volume ratios, including 1:3 (A), 1:5 (B), 1:10 (C), and 1:50 (D) at 25 °C. The concentration of hemin was 0.5 mg/mL. **Figure S3.** TEM images of hemin nanoparticles prepared by dialyzing for five days with various hemin/water volume ratios, including 1:3 (A), 1:5 (B), 1:10 (C), and 1:50 (D) at 25 °C. The concentration of hemin was 0.5 mg/mL. **Figure S4.** TEM images of hemin nanoparticles prepared by dialyzing for three days with the hemin/water volume ratio of 1:10 at various temperatures, including 4 °C (A) and 25 °C (B). The concentration of hemin was 0.5 mg/mL. **Figure S5.** UV–Vis spectrum of free hemin. The free hemin was dissolved in acidified aqueous acetone solution. (DOC 1146 kb)

## Abbreviations

DLS: Dynamic light scattering
FTIR: Fourier transform infrared
TEM: Transmission electron microscopy
XRD: X-ray diffractogram
