# Supplementary material for: Preparation and Characterization of Tadpole- and Sphere-Shaped Hemin Nanoparticles for Enhanced Solubility
Source: Nanoscale Res Lett. 2019 Feb 6;14:47. doi: 10.1186/s11671-019-2880-7 (PMC6365575; doi:10.1186/s11671-019-2880-7)
Supplement: Supplementary file 1 — Figure S1. TEM images of hemin nanoparticles prepared by dialyzing for one day with various hemin/water volume ratios, including 1:3 (A), 1:5 (B), 1:10 (C), and 1:50 (D) at 25 °C. The concentration of hemin was 0.5 mg/mL. Figure S2. TEM images of hemin nanoparticles prepared by dialyzing for two days with various hemin/water volume ratios, including 1:3 (A), 1:5 (B), 1:10 (C), and 1:50 (D) at 25 °C. The concentration of hemin was 0.5 mg/mL. Figure S3. TEM images of hemin nanoparticles prepared by dialyzing for five days with various hemin/water volume ratios, including 1:3 (A), 1:5 (B), 1:10 (C), and 1:50 (D) at 25 °C. The concentration of hemin was 0.5 mg/mL. Figure S4. TEM images of hemin nanoparticles prepared by dialyzing for three days with the hemin/water volume ratio of 1:10 at various temperatures, including 4 °C (A) and 25 °C (B). The concentration of hemin was 0.5 mg/mL. Figure S5. UV–Vis spectrum of free hemin. The free hemin was dissolved in acidified aqueous acetone solution. (DOC 1146 kb) [file 11671_2019_2880_MOESM1_ESM.doc]

Supporting Information
Preparation and [characterization](../../../../D:/%25E6%259C%2589%25E9%2581%2593/Dict/7.5.1.0/resultui/dict/javascript:%3B) of tadpole- and sphere-shaped hemin nanoparticles for enhancing solubility

Jie Yang, Liu Xiong, Man Li, Junxia Xiao, Xin Geng, Baowei Wang, Qingjie Sun*

College of Food Science and Engineering, Qingdao Agricultural University (Qingdao,
Shandong Province, 266109, China)
*Correspondence author (Tel: 86-532-88030448, e-mail: phdsun@163.com)
College of Food Science and Engineering, Qingdao Agricultural University, 266109, 700
Changcheng Road, Chengyang District, Qingdao, China.


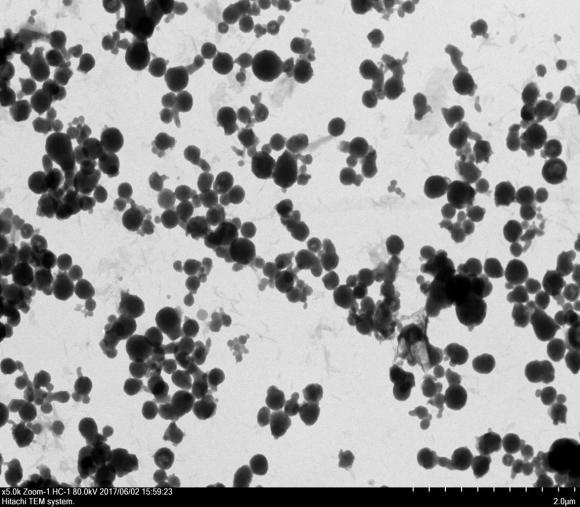


**200 nm**


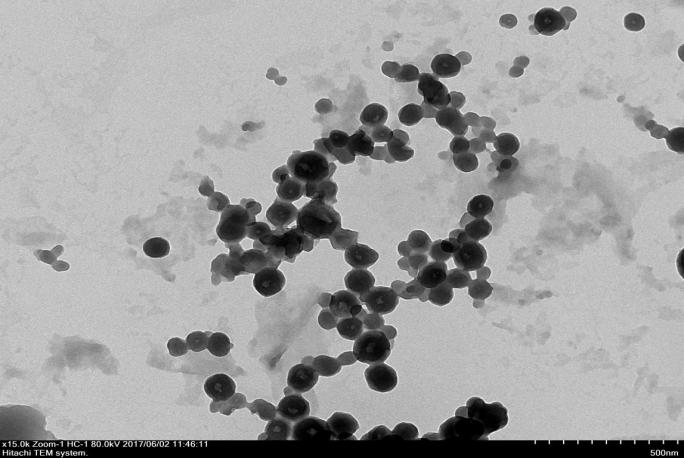


**200 nm**

B

A


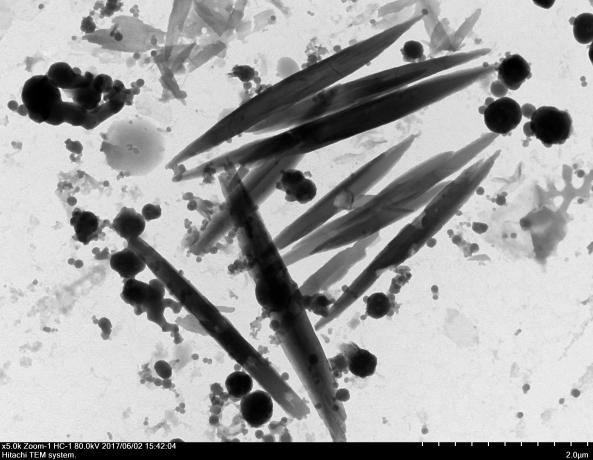


**200 nm**

D


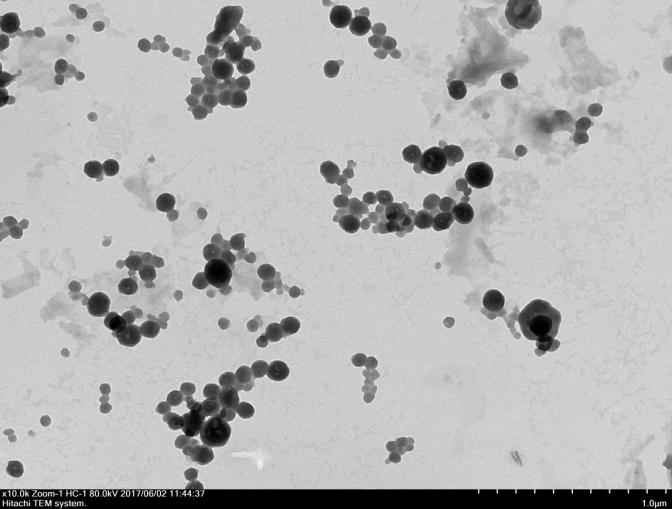


**200 nm**

C

Figure S1. TEM images of hemin nanoparticles prepared by dialyzing for one day with various hemin/water volume ratios, including 1:3 (A), 1:5 (B), 1:10 (C), and 1:50 (D) at 25 ºC. The concentration of hemin was 0.5 mg/mL.


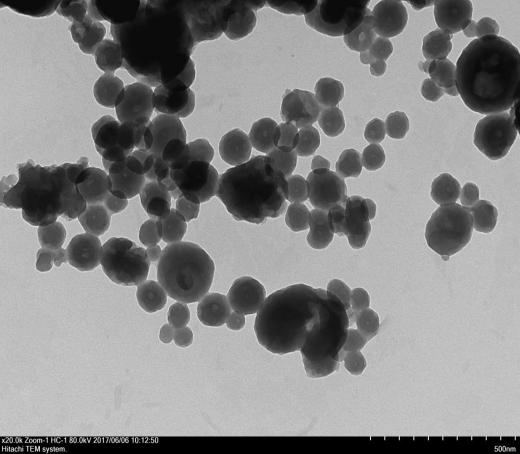


**200 nm**

A


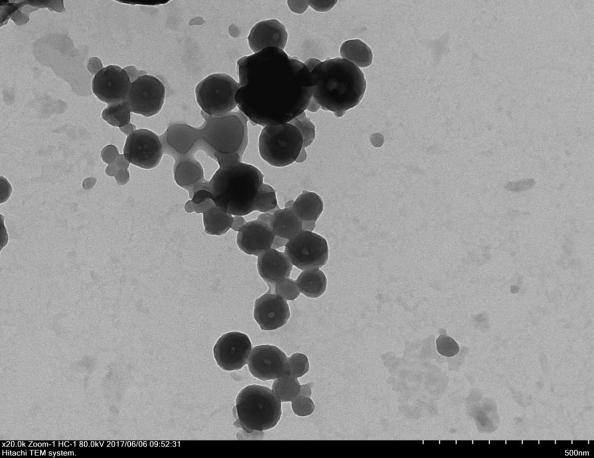


**200 nm**

B


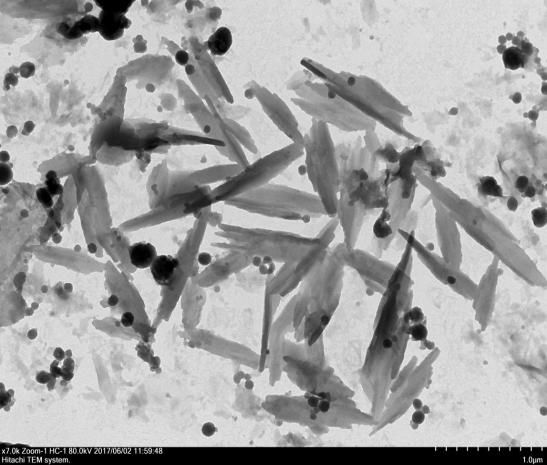


**200 nm**

D


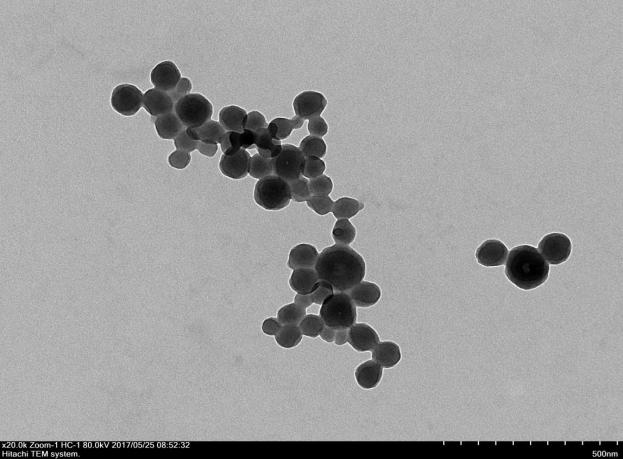


**200 nm**

C

Figure S2. TEM images of hemin nanoparticles prepared by dialyzing for two days with various hemin/water volume ratios, including 1:3 (A), 1:5 (B), 1:10 (C), and 1:50 (D) at 25 ºC. The concentration of hemin was 0.5 mg/mL.


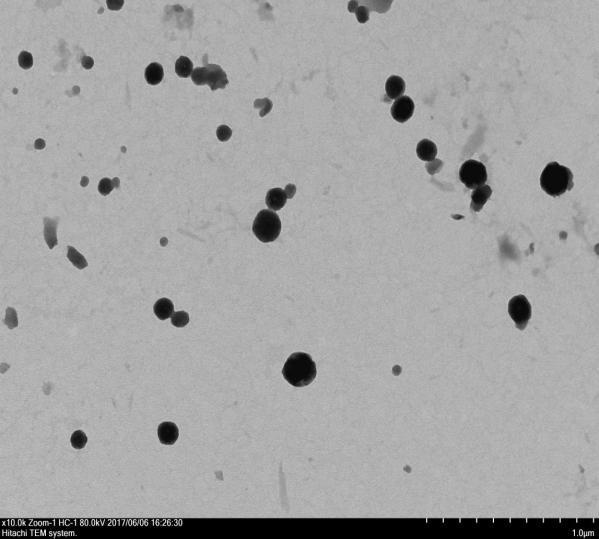


**200 nm**

A


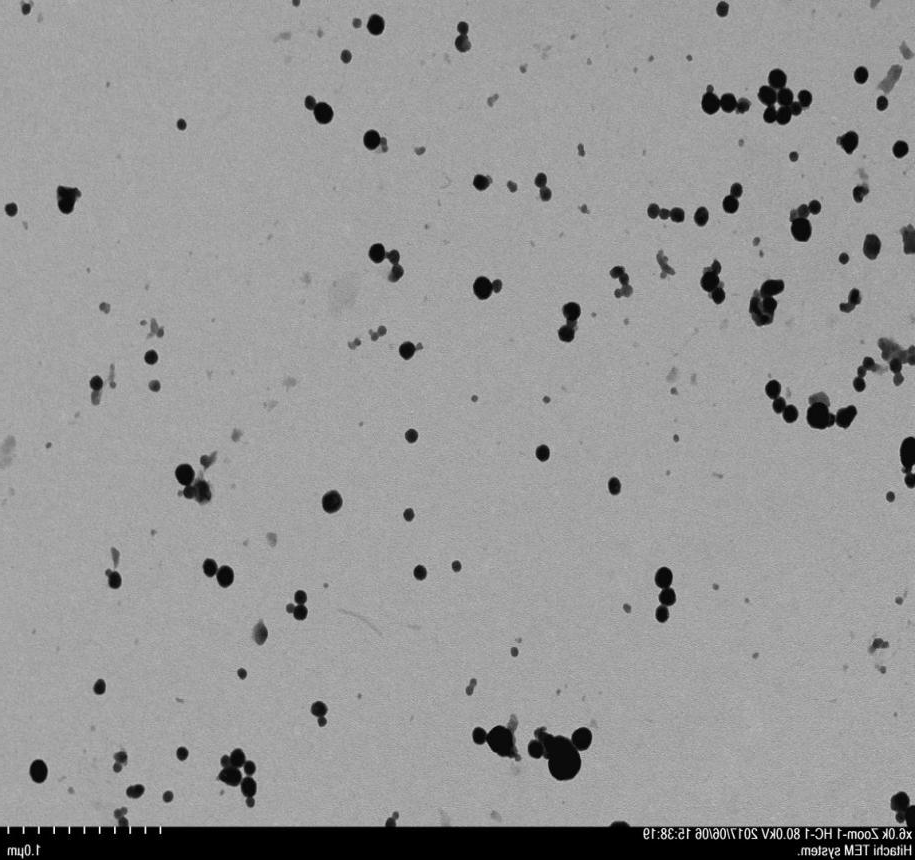


**200 nm**

B


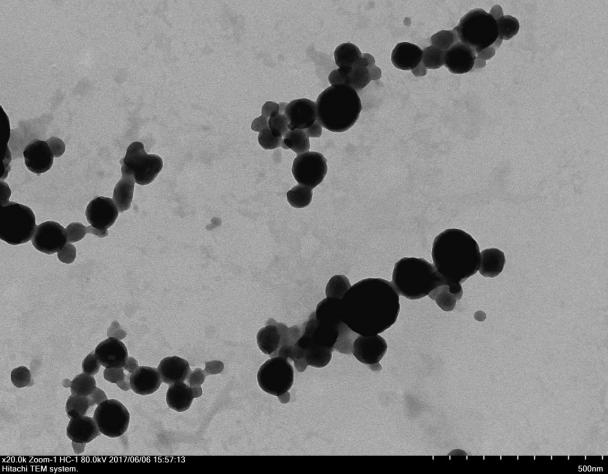


**200 nm**

D


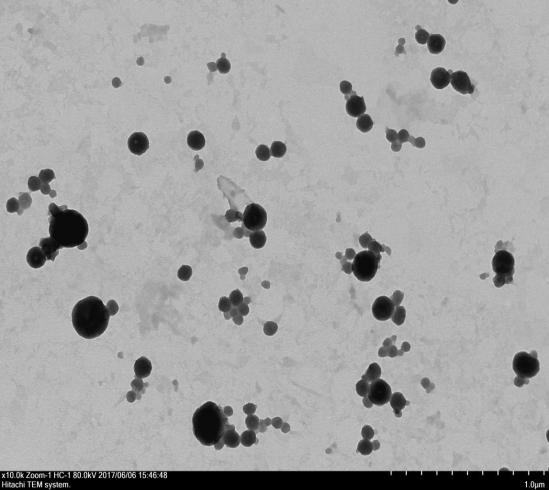


**200 nm**

C

Figure S3. TEM images of hemin nanoparticles prepared by dialyzing for five days with various hemin/water volume ratios, including 1:3 (A), 1:5 (B), 1:10 (C), and 1:50 (D) at 25 ºC. The concentration of hemin was 0.5 mg/mL.


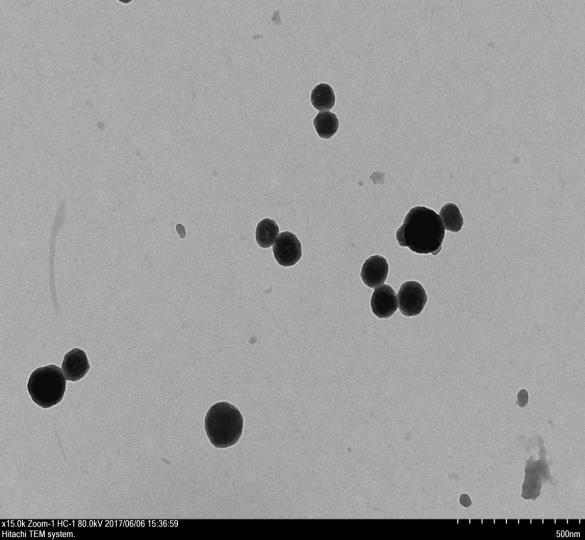


**200 nm**

A


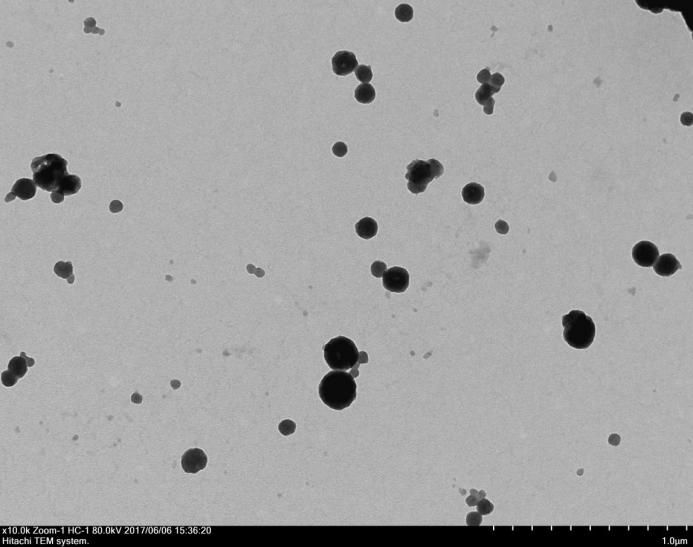


**200 nm**

B

Figure S4. TEM images of hemin nanoparticles prepared by dialyzing for three days with the hemin/water volume ratio of 1:10 at various temperatures, including 4 ºC (A) and 25 ºC (B). The concentration of hemin was 0.5 mg/mL.

Figure S5. UV-Vis spectrum of free hemin. The free hemin was dissolved in acidified aqueous acetone solution.
